# QOI1/338: Evaluation of Continuing Medical Education on the Internet

**DOI:** 10.2196/jmir.1.suppl1.e95

**Published:** 1999-09-19

**Authors:** M.W Peterson

**Affiliations:** ^1^University of Iowa, Iowa CityUSA

**Keywords:** Continuing Education, Computer

## Abstract

**Introduction:**

Continuing education has been a component of physician professional activity for over 500 years. The Internet, a powerful new communication system, offers new and novel tools for providing continuing medical education (CME) to healthcare professionals. However, it is not yet clear who will be providing the content or whether universities and university medical schools will utilize the new technology. Furthermore, few standards exist for controlling and measuring content quality on the Internet.

**Methods:**

To begin to address these questions and provide a picture of current Internet CME, 205 unique Internet host computers offering CME information were identified using standard search engines. The identified sites were reviewed for sponsoring organization, type of information, and quality using standards for quality control proposed by the JAMA.

**Results:**

University-sponsored Internet sites were the most common sites providing any CME information (44%). The next most common sites were commercial sites (26%). However, most (75%) university-sponsored sites offered only schedule information for traditional CME courses. By contrast, 42% of commercial sites offered on-line CME. Thus, in absolute numbers, more commercial sites than university sites offer on-line CME content. When gauging quality using the JAMA criteria, between 60 and 80% of the sites offering on-line CME meet the proposed standards for quality control. Of note, only 10% of the sites provide any information on content peer review. University sites performed slightly better than commercial sites in meeting these standards. Formal CME credits cost $15/hour on average; however, University sites charged significantly more than commercial sites. This difference was completely explained by the fact that nearly 50% of the commercial sites did not charge for CME and only 20% of the University sites provided free CME credit.

**Discussion:**

The Internet is beginning to provide opportunities for formal physician CME on-line. However, more of this CME is being provided by commercial sites than by university sites. The quality of the sites is not very high with only approximately 75% of the sites meeting a minimal standard for quality. These data suggest that universities may be losing their traditional leadership in CME using the new Internet medium. As healthcare professionals, we need to be concerned about the growing involvement of commercial sites in providing CME. These sites may have a market agenda in providing information. This concern is further supported by the fact that half of the commercial sites providing CME do not charge for the CME credits. Physicians need to remain vigilant in accepting information provided on-line, and universities need to reassert their traditional strengths in providing CME in this new medium.

